# A case report of recurrent membranoproliferative glomerulonephritis after kidney transplantation due to ventriculoatrial shunt infection

**DOI:** 10.1186/s12882-019-1472-1

**Published:** 2019-08-05

**Authors:** Linus A. Völker, Katharina Burkert, Niklas Scholten, Franziska Grundmann, Christine Kurschat, Thomas Benzing, Jürgen Hampl, Jan Ulrich Becker, Roman-Ulrich Müller

**Affiliations:** 1 Department II of Internal Medicine and Center for Molecular Medicine Cologne University of Cologne, Faculty of Medicine and University Hospital of Cologne, Uniklinik Köln, Kerpener Str. 62, 50937 Cologne, Germany; 2 Department of Neurosurgery, University Hospital Cologne, Kerpener Str. 62, 50937 Cologne, Germany; 3 Institute of Pathology, University of Cologne, Cologne, Germany

**Keywords:** Shunt nephritis, Membranoproliferative glomerulonephritis, Kidney transplantation, Recurrence

## Abstract

**Background:**

Transplant failure requires the consideration of numerous potential causes including rejection, acute tubular necrosis, infection, and recurrence of the original kidney disease. Kidney biopsy is generally required to approach these differential diagnoses. However, the histopathological findings on their own do not always lead to a definite diagnosis. Consequently, it is crucial to integrate them with clinical findings and patient history when discussing histopathological patterns of injury. The histopathologic finding of a membranoproliferative glomerulonephritis (MPGN) is one of the most challenging constellations since it does not refer to a specific disease entity but rather reflects a pattern of injury that is the result of many different causes. Whilst MPGN is occasionally classified as immune complex mediated, careful evaluation usually reveals an underlying disorder such as chronic infection, plasma cell dyscrasia, complement disorders, and autoimmune disease.

**Case presentation:**

We describe the case of a 43-year-old woman who was referred to us because of a slowly rising serum creatinine 4 years after kidney transplantation. As in the native kidney, the biopsy revealed an MPGN pattern of injury. The cause of this finding had not been established prior to transplantation leading to a classification as idiopathic MPGN in the past. Further workup at the time of presentation and allograft failure revealed chronic infection of a ventriculoatrial shunt as the most probable cause.

**Conclusion:**

This case underlines the fact that MPGN is not a disease but a histopathological description. Consequently, the causative disorder needs to be identified to avoid kidney failure and recurrence after transplantation.

## Background

**Fig. 1:**
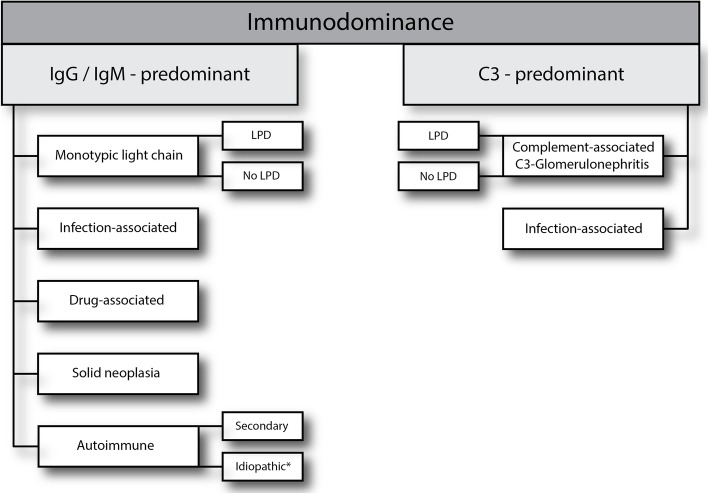
Differential diagnosis of endocapillary proliferative glomerulonephritis with subendothelial electron-dense deposits and membranoproliferative GN type I or III. Histopathological distinction into IgG/IgM-dominant forms and C3-dominant forms can help in the differential diagnosis. Note that light chain restriction may become apparent even in seemingly C3-dominant forms only after protease digestion [16]. The final diagnosis into the listed entities should always consider clinical findings, serum and urine tests for monoclonal gammopathy, complement serology, and complement genetics. Note that infection-associated glomerulonephritis can present histologically histology indistinguishable from C3-GN. C3-GN can be defined by its pathogenesis as mediated through complement dysregulation. The most common causes are hereditary or due to LPD-associated or idiopathic inhibitory autoantibodies, some of them presenting as C3-nephritic factors. Cryoglobulinemia falls into either the autoimmune-, LPD- or infection-associated categories in the left arm. Transplant glomerulopathy as a form of chronic antibody-mediated rejection may or may not show mesangial and subendothelial immune complexes, usually very few in number and [9] is thus not depicted in this scheme. Also not listed are TMA and associated lesions which are distinguished from MPGN or endocapillary proliferative GN by the absence of immune-complex-like electron dense deposits. * - Idiopathic is a diagnosis of exclusion, and the proportion of cases labelled as such is expected to shrink further with careful clinical assessment and our growing body of knowledge

Allograft failure can be caused by numerous differential diagnoses and usually requires kidney biopsy to confirm the actual pathology. Since one of these is recurrence of the disease leading to failure of the native kidneys, a lack of knowledge regarding this point adds to the challenge for nephrologists and nephropathologists. Even in patients in whom the native kidneys were biopsied, the histopathological diagnosis does not automatically lead to a clinical diagnosis. Most commonly, this is the case if the histopathological pattern of injury can be caused by numerous different clinical entities and if the underlying disease is generally rare. MPGN is such a finding with a number of different disorders – most of which are very rare – that can cause the same histopathological picture (Fig. 1) [1]. With chronic infections being one of the possible causes, shunt-nephritis may not be the first thought of many clinicians due to its low incidence. This holds true especially in the setting of transplant failure. Shunt-nephritis refers to a form of glomerulonephritis that is often characterized by an MPGN pattern on the histological level associated with polyclonal immune complexesin subacute and chronic courses while acute courses may present as endocapillary or extracapillary diffuse-proliferative, mesangiocapillary, and mesangial lesions [2–5].

In the case presented here, MPGN is the consequence of immune-complex deposition induced by chronic infection. Chronic infections of a ventriculoatrial or –peritoneal shunts are most commonly caused by colonization with slowly-growing bacteria of the skin flora, e.g. staphylococci [3, 4]. Many cases of these infections are characterized by a subacute to chronic clinical course (such as recurrent fever, hepatosplenomegaly, and cerebral symptoms). However, very slow chronic and oligosymptomatic courses have also been described [5, 6]. Apart from the unspecific clinical picture, problems in cultivating slowly growing bacteria from blood and liquor cultures make both the diagnosis and a targeted anti-infective therapy difficult. Consequently, these cases are prone to be misinterpreted and diagnosed late when immunological complications such as shunt nephritis have already manifested. However, if recurrent MPGN threatens graft survival, identification of this rare cause is crucial since this problem can be addressed specifically by removing the shunt material and treating with antibiotics according to the resistogram. Finding a way to remove all artificial materials and solving the problem causing hydrocephalus in such a patient without reimplantation of a new shunt are of utmost importance. Here, we present the exemplary case of a young woman illustrating both the differential diagnosis of diseases underlying recurrent MPGN in kidney transplants including the algorithm to identifying shunt nephritis as the causative disorder and the interdisciplinary approach to solving the problem.

### Timeline

**Table:** 

Year	Event
1986	Diagnosis of hydrocephalus Placement of ventriculoperitoneal shunt
1992	Placement of ventriculoatrial shunt
2004	Diagnosis of membranoproliferative glomerulonephritis
2004–2008	Immunosuppressive therapy with cyclosporine and steroids
2008	Placement of a AV fistula as dialysis access
2009–2012	Hemodialysis
Feb 2012	Kidney transplantation (cadaveric donor)
Mar 2012	Allograft biopsy: No sign of rejection
Jun 2012	Allograft biopsy: Borderline T cell-mediated rejection, steroid pulse
Jan 2016	Allograft biopsy: Recurrence of MPGN VP−/VA-shunt-removal Endoscopic ventriculostomy
Feb 2016	Surgical removal of remaining shunt fragments Transcutaneous-endovascular retrieval of VA-shunt fragments
Oct 2018	Improved allograft function, complete resolution of hematuria and proteinuria

## Case presentation

**Fig. 2:**
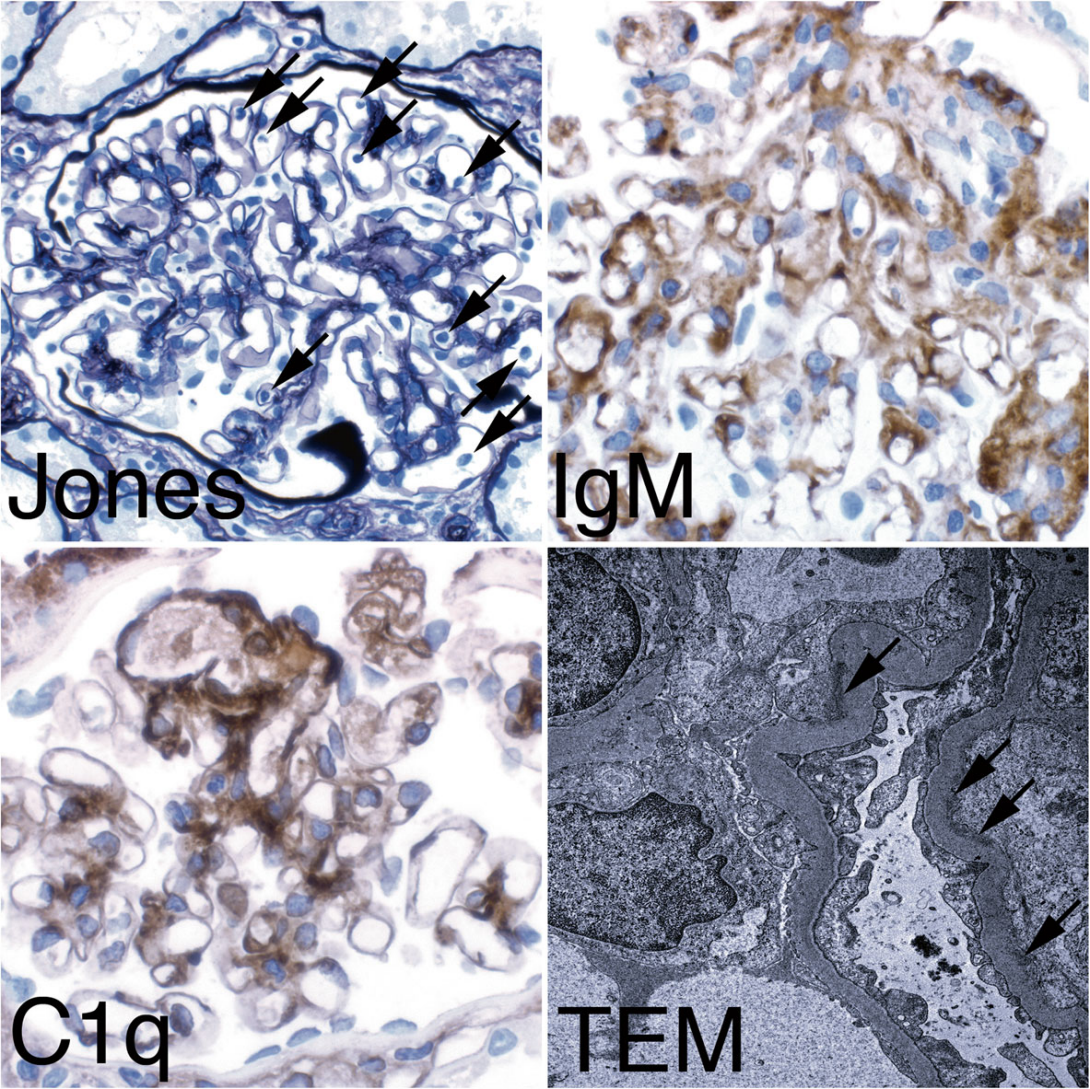
Histopathological findings in the kidney transplant biopsy. The top left (Jones) shows increased numbers of mononuclear cells in the glomerular capillaries (arrows). Jones silver stain, original magnification × 400. The top right (IgM) shows IgM dominant deposits along the glomerular basement membrane in brown. IgA and IgG were virtually negative. Immunoperoxidase, original magnification × 630. The bottom left shows strong C1q co-deposition along the glomerular basement membrane in brown. Immunoperoxidase, original magnification × 630. The bottom right shows subendothelial electron dense immune complex deposits on the inner side of the glomerular basement membranes (arrows), typical for endocapillary proliferative GN or MPGN type I. Original magnification × 10,000

A 43-year-old Caucasian woman, who had undergone cadaveric kidney transplantation 4 years earlier, was referred to us due to a creeping rise in serum creatinine. Her medication included cyclosporine, mycophenolate-mofetil, prednisone, omeprazole, and oral sodium bicarbonate. She had been on hemodialysis for 3 years before receiving the allograft. To determine the diagnosis leading to end-stage renal disease, a native kidney biopsy had been performed 12 years ago showing MPGN in histopathology. According to her file, a further diagnostic workup back then had not revealed any signs of an underlying condition leading to the classification as idiopathic MPGN. However, due to the long time passed, no data was available regarding the details of the exams that had been performed. Upon presentation, the patient reported malaise, elevated body temperature and shivering when combing her hair or working over-head. She had been experiencing occasional milder episodes of unclear infection during the last decades. Aside from an elevated arterial pressure of 165/85 mmHg, the physical examination was unremarkable. We conducted a complete laboratory blood analysis, which showed a serum creatinine of 183 μmol/L (eGFR CKD-EPI 24 ml/min; best creatinine after transplantation: 92.3 μmol/L, eGFR 56.1 ml/min), a mildly elevated c-reactive protein of 275 nmol/L (28.9 mg/L), a significantly increased procalcitonin (11.1 μg/L), low C3 complement (0.78 g/L), and cyclosporine levels within the target range. The initial urine analysis revealed microhematuria with mild albuminuria of 49 mg/g creatinine. Repeated blood and urine microbial cultures remained sterile. Ultrasound examination of the transplant kidney revealed a normal size without parenchymal or vascular abnormalities. Kidney biopsy showed an active endocapillary proliferative glomerulonephritis - consistent with an early stage of recurrent membranoproliferative glomerulonephritis type I (Fig. 2) with electron dense subendothelial deposits lacking significant glomerular basement membrane splitting. Deposits were positive for IgM, C1q and C4d, with IgA, IgG and C3c virtually negative, excluding a C3-glomerulopathy. Serological studies for human immunodeficiency virus, hepatitis virus, Epstein-Barr virus, cytomegalovirus, parvovirus B19 showed no signs of active disease. Monoclonal gammopathy was excluded by serum and urine immuneelectrophoresis and a serum free light-chain assay. Transesophageal echocardiography demonstrated absence of endocarditic vegetations. Other infectious foci were excluded by sonography and clinical examination. DsDNA-antibodies, anti-neutrophil antibodies, and donor-specific antibodies were not detected. We found anti-nuclear antibodies (subtype SS-A / Ro-60) with a titer of 1:320. Apart from the transplant dysfunction there were no clinical signs of specific autoimmune diseases. The patient did not report recent travels to foreign countries. A ventriculoperitoneal shunt (VP) had been placed at the age of 14 due to congenital aqueductal stenosis and hydrocephalus. At the age of 21 the VP shunt had been switched to a ventriculoatrial shunt (VA) due to recurrent abdominal pain. It had not been possible to recover remnants of the VP-shunt, which had remained in situ.

Shivering on working over-head, recurrent laboratory signs of inflammation, and excluding other causes of MPGN were the clinical cues that led us to suspect a chronically infected VA-shunt as trigger of transplant failure.

To assess the feasibility of shunt removal, we conducted an MRI of the cerebrospinal fluid circulation and interspaces. Imaging revealed a triventricular hydrocephalus with aqueductal stenosis but patent interventricular foramina. Thus, the patient was subjected to endoscopic ventriculocisternostomy and placement of an external liquor drainage, which was removed as soon as the ventriculocisternostomy had proven to drain liquor efficiently. VP- and VA-shunt material was removed surgically. Due to extensive calcification of the VA-shunt-fragment in the superior VC, a 5 cm fragment could not be mobilized manually and had to be removed interventionally via femoral vein access, thus obviating open cardiothoracic surgery. The calcifications remained in place. Despite negative blood and cerebrospinal fluid (CSF)-cultures, microbial analysis detected *Staphylococcus hominis* and *epidermidis* on the VP-shunt and *Staphylococcus epidermidis* on the atrial catheter fragment. All recovered intracranial material remained culture-negative. In accordance with the resistogram, flucloxacilline (3 g IV QID) was administered for 14 days intravenously; antibiotic therapy was continued for another ten days using amoxicillin/clavulanic acid (500/125 mg PO TID). Kidney function began to improve immediately after shunt removal, serum creatinine reached 119.8 μmol/L (eGFR 40 ml/min) 10 weeks after shunt extraction. Hematuria improved in parallel. There were no new episodes of fever and shivering while inflammation parameters returned to normal. The patient did not show any neurological symptoms and was discharged after a course of 39 days. Ever since completion of antibiotic therapy our patient has been free of any renal or systemic symptoms of infection at the last follow-up approximately 1.5 years after shunt removal. Allograft function has improved since discharge (last eGFR 46 ml/min). With complete resolution of hematuria and microalbuminuria, the patient shows no remaining signs of glomerular disease.

## Discussion and conclusion

This case clearly illustrates that MPGN must not be considered a disease entity but a histopathological pattern. This pattern can be caused by numerous underlying diseases, many of which are rare by themselves [7]. In transplant kidneys even antibody-mediated rejection and hepatitis C-virus infection can show this pattern [8, 9]. In our patient, disease recurrence was likely. Yet, the nature of the underlying disease had not been elucidated prior to transplantation and needed to be established to direct therapy. Etiology of MPGN can be grouped into 1. polyclonal immune-complex mediated, 2. monoclonal immunoglobuline deposition related, 3. C3-dominant cases and 4. as a result of primary endothelial injury. Workup of patients with an MPGN (or an endocapillary proliferative glomerulonephritis) pattern after exclusion of C3-glomerulopathy requires exclusion of chronic infections, autoimmune disease, adverse drug effects, and hematological neoplasia (Fig. 1). In the course of this workup, it is crucial that all rare causes are excluded. The term idiopathic or primary MPGN mainly reflects incomplete workup missing the underlying etiology. Shunt nephritis as one of these rare infectious causes falls into the category of MPGN related to polyclonal immune-complexes and arises through immune complex chronic antigenemia and polyclonal immune complex deposition in the glomerular tuft, classically presenting with IgM-dominant immune complex deposits like in our case [7, 10]. Colonization with *Staphylococcus epidermidis* is believed to be the most frequent cause of shunt nephritis although multiple other pathogens have been identified [6, 11–14]. Similar processes can take place in case of bacterial endocarditis. Shunt nephritis can manifest as diffuse endocapillary proliferative glomerulonephritis similar to poststreptococcal GN, as mesangioproliferative GN, or as MPGN [3]. The severity of GN predominantly depends on the duration of antigenemia and chronic infection. Most cases have a subacute course with neurological symptoms and rapid decline of kidney function so that early shunt removal is critical. If complete shunt removal is possible, renal prognosis is favorable [15].

The case presented clearly shows the importance of being aware of shunt nephritis as a differential diagnosis of diseases underlying MPGN or endocapillary proliferative GN. In hindsight, it is highly likely that the MPGN in the patient’s native kidneys was in fact shunt nephritis. However, due to an equivocal clinical picture and a lack of alternative therapeutic options at that time, the shunt had remained in place. Differently from most cases reported so far, our patient presented with a rather chronic course of the disease - as indicated by persistently negative blood and CSF-cultures and lack of neurological symptoms – which may be the consequence of slowly growing bacteria with low pathogenicity.

The remaining calcifications in the superior VC may still harbor pathogens causing persistent antigenemia potentially leading to immune complex formation and deposition. However, in contrast to artificial shunt material, which is predisposed to staphylococcal biofilm formation, calcifications undergo constant turnover so that innate host immune mechanisms may clear remaining pathogens. The optimal duration of antibiotic therapy after shunt removal remains to be determined.

## Data Availability

The data set used in this study has been reported in the case presentation. Additional clinical data and laboratory values are available upon request from the corresponding author.

## Abbreviations

ANCA: Anti-neutrophil cytoplasmic antibody
CMV: Cytomegalovirus
CSF: Cerebrospinal fluid
dsDNA: Double stranded desoxyribonucleic acid
EBV: Epstein-Barr virus
GN: Glomerulonephritis
MPGN: Membranoproliferative glomerulonephritis
MRI: Magnetic resonance imaging
TMA: Thrombotic microangiopathy
VA: Ventriculoatrial
VP: Ventriculoperitoneal
